# Is There Any Thing New under the Sun?

**Published:** 1874-08

**Authors:** 


### IS THERE ANYTHING NEW UNDER THE SUN?

JACKSONVILLE, FLA., June 26th, 1874.

*Editor Atlanta Medical and Surgical Journal:* In your article upon intestinal obstructions, in the June number of your valuable journal, I see that you claim to be the originator of the method of relieving cases of intussusception of the bowels by copious enemas of tepid water. I enclose a copy of a note from Dr. A. S. Baldwin, of this city, showing that he has been using the method for many years.

JACKSONVILLE, FLA., June 25th, 1874.

*Dr. C. Drew:* SIR: In reply to your inquiry last evening, as to when I commenced the use of large injections by the pump for the relief of intussusception of the bowels, and where my account of my mode of treatment was first published, I will state that, I first tried the plan in September, 1848, and you will find in the *American Journal of Medical Sciences,* for October, 1852, page 568–9, the extract from my letter, giving an account of this operation, which had been tried four years previous. The plan there described was entirely original with myself, never having heard or read of it before.

Only a short time previous to the adoption of this method of relief, I had lost a strong and generally healthy man, in whose case I had used all the methods and appliances recommended in the books and journals that I could find, but without any benefit; but I think the injections with the long tube did harm, by further distending the bowels. In the next case I went through the same routine, and with no benefit. I came to the conclusion that it was necessary to distend the bowel below the point of obstruction, and thereby draw out the invaginated portion of the intestine. This it did in this instance, and probably in a hundred cases since, where this plan has been tried, several of which cases were placed in my charge by my professional brethren, who were aware that I had relieved this disease in many instances. I have been able to relieve all cases which have come under my care thus far since. I do not wait, however, to see if it is going to be a desperate case—under the old plan of treatment—but at an early stage, make the application and furnish prompt relief.

I was induced, at the earnest solicitation of some of my medical brethren and my patrons, to lay this plan before the medical profession, as being valuable information for general dissemination.

In the summer of 1857, while on my way North, the Hon. Col. Benton was very sick with this disease, and all the ordinary methods had failed to procure relief. At the solicitation of some of my Florida friends, who happened to meet me there, I gave a description of my mode of procedure, which, I understood, was applied, and with success; and when I arrived in New York, the papers announced that the Colonel had been relieved by a new operation.

I have used this operation now for over twenty-five years, with success; and if any one had used it before me, I am not aware of the fact. Respectfully yours,

A. S. Baldwin, M.D.

This I think, goes to prove that Dr. Baldwin is the originator of the "method of relieving invagination of the intestine by copious injections of tepid water." I have seen him use the method, injecting water to the fully capacity of the bowel. During the operation, the patient who had previously vomited only small portions of fæcal matter, vomited clear water freely, which could only have entered the stomach by finding its way from the rectum. The method has long been known here as "Dr. Baldwin's method," and has frequently proved successful when everything else had failed.

Respectfully, yours,

C. Drew, M.D.

We publish the letter of our correspondent with pleasure, not alone as an act of justice and courtesy, but that our readers may become acquainted with the history of an important discovery, whosever it may be—and it matters little whose. The fact itself is *the thing*, and not the discoverer of it.

If we consider the countless number of practitioners of the medical art who constitute the immense cordon of ministering spirits that encircles the entire globe; if we allot to each one his fair proportion of suffering, perishing mortality, is it matter of surprise that two, or ten, or fifty of this countless host should, independently and simultaneously, smell of the poppy-blossom and nod as the consequence? Might not one in China, another in Europe, and yet a third in America, without intervention of either the mail or the telegraph, be seized with a boyish impulse to test the gustatory qualities of the castor bean? Suppose each of the three should be seized with unwonted intestinal peristalsis, and all should make the common discovery that the bean is purgative, would the Attorney-General be justified in bringing an action for scientific larceny in such case?

Does anybody believe that McCormick, who pocketed his millions, was really the inventor of the great *reaper* which has rendered possible, in our day, the gigantic West, with its teeming millions of population? If so, will such an one but turn to the old Edinburgh Encyclopædia and find there the initiatory ideas duly set forth. Does anybody believe that either Horace Wells or Morton, or Jackson, really discovered anæsthesia, that stupendous wonder of our day? If so, let him turn to the pages of the immortal Shakespeare, in his inimitable play of Romeo and Juliet, and acknowledge his error.

If one man may take sugar in his grog and another should altogether eschew the sweetening, it is no matter of surprise that some should elect to sweeten their calomel and others to take it *straight*; and we respectfully submit that neither the grog nor the calomel, whether sweetened or straight, ought to be entertained by the Commissioner of Patents as the individual property of any one man. Necessity is the mother of invention, says the proverb; and from it, by very natural induction, we may draw the inference that wherever the fruitful dame is found there we may look for her progeny.

Thackeray, in his "Snobs," puts it thus: "We have all read a statement that when the times and necessities of the world call for a man, that individual is found. Thus at the French Revolution, when it was requisite to administer a corrective dose to the nation, Robespierre was found a most foul and nauseous dose indeed, and swallowed most eagerly by the patient, greatly to the latter's ultimate advantage: thus, when it became necessary to kick John Bull out of America, Mr. Washington stepped forward, and performed that job to satisfaction: thus, when the Earl of Aldborough was unwell, Professor Holloway appeared with his pills, and cured his Lordship, as per advertisement, etc. Numberless instances might be adduced to show that when a nation is in great want, the relief is at hand, just as in the pantomime (that microcosm) where when the *clown* wants anything—a warming-pan, a pump-handle, a goose, or a lady's tippet—a fellow comes sauntering out from behind the side-scenes with the very article in question."

Years ago, Mr. I. Baker Brown, the London gynæcological surgeon, having by much study, experiment, and diligent labor, perfected, as he supposed, a new and most promising operation for the cure of intra-uterine fibroids, with light and joyous heart jumped upon the railway train for Edinboro, bearing in his hands a few turnips from the market stall, that he might demonstrate his discovery satisfactorily to his friend, Sir James Simpson. When the case had been fully presented, Simpson produced a musty tome in antiquated, parchment cover, of which Brown had not previously so much as heard the name of the author even, and read to him the description of an operation, in essence, the same. Was Baker Brown a discoverer? Certainly so—he was the re-discoverer of a lost art, which but for him might have remained forever buried from the world.

In the museum of the Imperial Porcelain Factory, in the suburbs of Paris, there stood a colossal earthen jar of antique moulding. To-day there lives not upon the green earth the man who can duplicate that jar. Suppose to-morrow some fellow comes "sauntering out from behind the side-scenes" with its exact counterpart, is he a discoverer? Who can doubt it?

We have but little taste for controversy in questions of priority of medical discovery and invention. So the car of scientific progress moves onward, it matters little to the interested world who rides upon the top, or who may be crushed beneath its ponderous wheels. We have opened our columns quite freely for the ventillation of *saccharated calomel*, not, really, so much for the purpose of settling the question of priority, as that the attention of our readers might be fixed upon what we esteem a truly valuable combination, which was but little known and less appreciated; and, secondly, that they might reap a collateral harvest of valuable knowledge to be unearthed in the diligent search for precedents.

As regards the traversing of the entire alimentary canal with a liquid introduced *per rectum*, we have neither time nor inclination to contest any claim which may be made to prior discovery.

We have not at hand the authority referred to by our correspondent, nor are we disposed to search for it. The re-discovery, if it be such, is certainly new to us; it is new to the great mass of our readers. That its publication was timely and useful will not be denied; and if the objectionable passages we have pointed out in Flint's standard work upon Practice should be dropped, or suitably modified in coming editions of that valuable work, we will have achieved a practical result, which our predecessors may have deserved, but have not realized, and will have been amply rewarded for our labor.

